# Thymic Aging May Be Associated with COVID-19 Pathophysiology in the Elderly

**DOI:** 10.3390/cells10030628

**Published:** 2021-03-12

**Authors:** Weikan Wang, Rachel Thomas, Jiyoung Oh, Dong-Ming Su

**Affiliations:** 1 Cell Biology, Immunology, and Microbiology Graduate Program, Graduate School of Biomedical Sciences, University of North Texas Health Science Center, Fort Worth, TX 76107, USA; Weikan.Wang@unthsc.edu (W.W.); RachelThomas@my.unthsc.edu (R.T.); 2 Department of Pediatrics, University of Texas Southwestern Medical Center, Dallas, TX 75390, USA; Jiyoung.Oh@unthsc.edu; 3 Department of Microbiology, Immunology & Genetics, University of North Texas Health Center, 3500 Camp Bowie Blvd, Fort Worth, TX 76107, USA

**Keywords:** aged COVID-19 patients, aged thymus, thymic involution, role of T cells, immunopathology

## Abstract

Severe acute respiratory syndrome coronavirus 2 (SARS-CoV-2) caused the global pandemic of coronavirus disease 2019 (COVID-19) and particularly exhibits severe symptoms and mortality in elderly individuals. Mounting evidence shows that the characteristics of the age-related clinical severity of COVID-19 are attributed to insufficient antiviral immune function and excessive self-damaging immune reaction, involving T cell immunity and associated with pre-existing basal inflammation in the elderly. Age-related changes to T cell immunosenescence is characterized by not only restricted T cell receptor (TCR) repertoire diversity, accumulation of exhausted and/or senescent memory T cells, but also by increased self-reactive T cell- and innate immune cell-induced chronic inflammation, and accumulated and functionally enhanced polyclonal regulatory T (Treg) cells. Many of these changes can be traced back to age-related thymic involution/degeneration. How these changes contribute to differences in COVID-19 disease severity between young and aged patients is an urgent area of investigation. Therefore, we attempt to connect various clues in this field by reviewing and discussing recent research on the role of the thymus and T cells in COVID-19 immunity during aging (a synergistic effect of diminished responses to pathogens and enhanced responses to self) impacting age-related clinical severity of COVID-19. We also address potential combinational strategies to rejuvenate multiple aging-impacted immune system checkpoints by revival of aged thymic function, boosting peripheral T cell responses, and alleviating chronic, basal inflammation to improve the efficiency of anti-SARS-CoV-2 immunity and vaccination in the elderly.

## 1. Introduction

Currently, the global pandemic of coronavirus disease 2019 (COVID-19), caused by severe acute respiratory syndrome coronavirus 2 (SARS-CoV-2), poses a greater threat to elderly people than to children and young adults, as shown by a higher frequency of severe symptoms and mortality in elderly patients, while children and young adults usually present with mild disease [1,2]. Differences in clinical severity are likely associated with immune system age [3]. Both the innate and adaptive immune systems are involved in antiviral responses. Although the innate immune system responds early, adaptive antiviral immunity is specific and robust, lasting longer in combating viral infection and generating immune memory. Adaptive antiviral immunity primarily includes neutralization antibodies (Ab) [4] associated with B cells, and cellular (mostly T cell)-mediated anti-SARS-CoV-2 immunity [5,6,7,8]. Although specific Abs are important for an immunoprotective barrier by blocking free viral particles from entering host cells, T cells and NK (nature killer, containing both innate and adaptive immune features) cells are more powerful because they destroy virally infected cells, thereby terminating viral replication. Generally, T cell priming is a key factor for effective immunity and vaccination, since T cells act not only as killer cells, but also as helper cells. For example, CD8^+^ T cells with cytotoxic T lymphocyte (CTL) function conduct killing of virally infected cells. Mild COVID-19 patients exhibit more CD8^+^ CTL cells [7,8], while patients with severe disease have predominantly increased SARS-CoV-2-specific CD4^+^ T cells in their recovery-stage of the disease [7,8]. These differences imply that different T cell subsets have different roles in disease severity and outcome. CD4^+^ T helper cells support the B cell-mediated antibody-producing humoral response. Additionally, some act as regulatory cells either via cytokine secretion, such as CD4^+^ Th1 (T-helper 1) cells, which primarily produce interferon-γ (IFN-γ), tumor necrosis factor-α (TNF-α), etc., and Th2 cells, which primarily produce interleukin-(IL)-4, IL-10, etc., and Th17 cells (producing IL-17), or facilitate immunosuppression (via multiple mechanisms, including inhibitory cytokines), such as CD4^+^FoxP3^+^ regulatory T (Treg) cells. Th1-biased cellular immune responses typically direct the killing of the virus, while Th2-biased responses are usually associated with lung allergy in respiratory infections [9]. The roles of Treg cells reported during COVID-19 are thus far contradictory, either reportedly decreased [10,11] or relatively increased in COVID-19 patients with severe disease or/and lymphopenia [6,12,13]. The roles of Treg cells in COVID-19 patients should perhaps be assessed based on their physiological localization and disease stage. If increased Treg cells are in the lung during an inflammatory cytokine storm, this will probably be beneficial for the alleviation of the excessive immune response [14,15], but if increased Treg cells are present early in the disease, it could be detrimental to the establishment of effective antiviral immunity.

Age-related changes to the T cell immune system include three main characteristics: (1) immunosenescence: low immune response, due to restriction of the TCR repertoire diversity, coupled with an increased oligoclonal expansion of peripheral memory/senescent T cells; (2) established chronic inflammation in the elderly, termed inflammaging, which is partially due to increased self-reactive T cell-induced chronic self-tissue damage, in addition to pro-inflammatory somatic cellular senescence-associated secretory phenotype (SASP); (3) enhanced polyclonal Treg cell generation in the aged, atrophied thymus and Treg accumulation in the aged peripheral secondary lymphoid organs. Evidence shows that all these changes are mainly attributed to age-related thymic involution [16].

**Figure 1:**
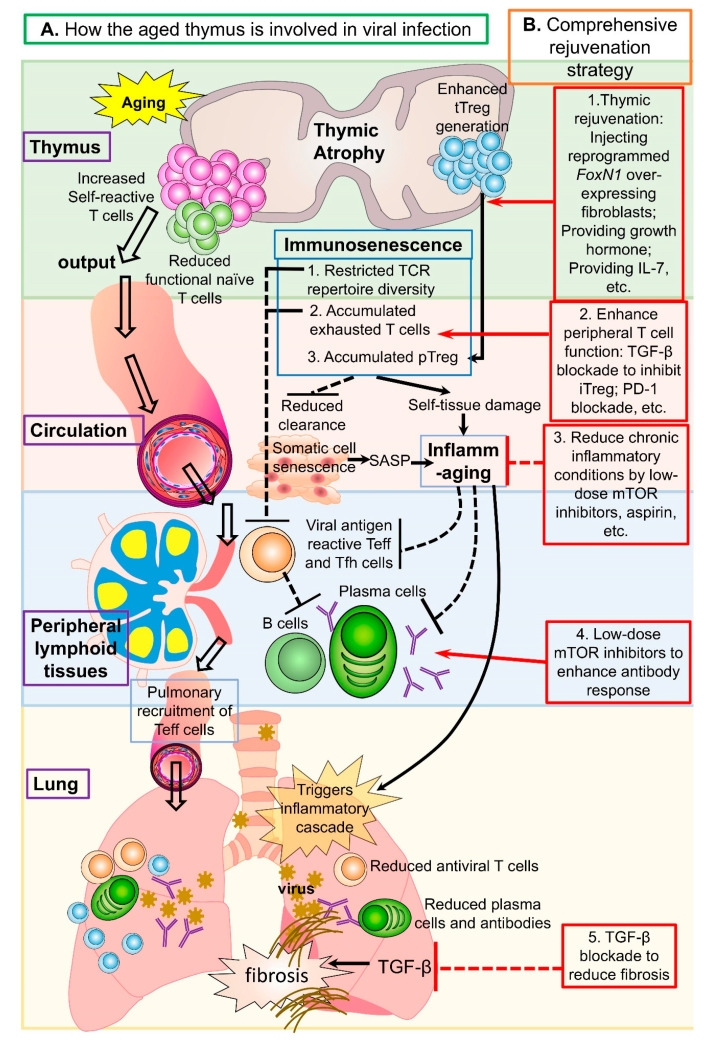
How the aged thymus is involved in viral infection and a proposed comprehensive rejuvenation strategy for enhanced antiviral immunity and vaccination efficiency. (**A**) Left panels show the T cell pathway from the thymus to the lung during respiratory viral infection, such as SARS-CoV-2, using arrows. Middle panels show how immunosenescence and inflammaging are detrimental to antiviral immunity. (**B**) Right panels (red boxes) are proposed rejuvenation checkpoints where the dotted red lines are inhibition or blockade and the solid red lines with arrows are promotion or enhancement.

Immunosenescence and inflammaging are high risk factors for severe COVID-19 in the elderly [1,2,17,18]. As age-related thymic involution contributes to immunosenescence and inflammaging (Figure 1A, Table 1 third column) [16], thymic function should also be considered as a potential player in aged populations versus young [19,20], and may also impact vaccination efficiency in the elderly. One indication that thymic function participates in COVID-19 disease severity has been reported, in which thymosin alpha-1 (T α 1, a synthetic thymic peptide) reduced the mortality of patients with severe COVID-19 [21], and a clinical trial with T α 1 to treat COVID-19 infection in elderly patients was approved (https://clinicaltrials.gov/ct2/show/NCT04428008 (12 January 2021)). Therefore, rejuvenation of aged thymic function in combination with an improvement in the pre-existing aged peripheral T cell microenvironment and inflammaging could improve protective immunity and efficient vaccination against viruses, including SARS-CoV-2, in the elderly.

In this review paper, we raise the hypothesis that thymic aging plays a potential role in clinical severity of aged COVID-19 patients based on aged T cell immune system features and the observed symptom disparities between children and young adults compared to elderly COVID-19 patients. Then, we address which components of the aged T cell system potentially contribute to COVID-19 pathophysiology in the elderly. Finally, we suggest several promising strategies for rejuvenating thymic function and reducing the peripheral basal inflammatory environment in order to boost antiviral immunity and vaccination efficacy in the elderly. Together, we provide an immunological perspective outlining possible implications of the thymus in SARS-CoV-2 infection in the elderly and provide insights into the potential contribution of the thymus to the clinical severity of COVID-19 pathology in young and aged patients.

## 2. Does Thymic Aging Play a Role in the Severity of Aged COVID-19 Patents?

Based on currently available evidence from the current COVID-19 pandemic, most cases present with mild respiratory distress symptoms, with only a few of cases having severe pneumonia [22]. Among the severe cases, the majority are adults with underlying health conditions and elderly individuals. Children and young adults exhibit less susceptibility to the disease than the elderly [19,23,24]. Although it is proposed that one reason for the reduced clinical severity in children is due to reduced expression of angiotensin-converting enzyme 2 (ACE-2) receptors, which is the key receptor needed for SARS-CoV-2 infection of epithelial cells of the host respiratory tract [25], the overall robustness of the immune system is also a key distinction between young and old individuals. Studying the unique characteristics of the immune system in children and young adults, including innate and adaptive components, will likely reveal the potential mechanisms needed to understand efficient antiviral immunity and vaccination in the elderly.

Changes in the aged immune system [26,27,28] result in anti-infection immune insufficiency (immunosenescence) and self/auto-immune enhancement (partially contributing to age-related chronic inflammation, i.e., inflammaging). One of the most obvious age-associated alterations in the aged immune system is the involution/atrophy of the thymus [16,29]. The thymus plays a key role in cellular immune function and it continuously develops undifferentiated thymocytes into functional naïve T cells throughout the lifetime to facilitate adaptive immunity. However, the thymus undergoes progressive physiological involution with age [30]. The involuted thymus exhibits reduced naïve T cell output, contributing to a restricted TCR repertoire with reduced ability to recognize neo-antigens, which results in increased susceptibility to infection. Meanwhile, the involuted thymus exhibits increased self-reactive T cell output due to defective negative selection, which results in increased self-reactivity associated with autoimmune proneness and inflammaging [16]. Additionally, as various types of coronaviruses are able to induce thymic involution, SARS-CoV-2 could also possibly damage thymus [20], which further deteriorate the functionality of aged thymus in T cell generation. Thus, we can assume that the decline in T cell immunity via thymic involution is potentially involved in the increased morbidity and mortality of COVID-19 in the elderly.

It is unclear how T cells are involved in SARS-CoV-2 infection [31]. However, lower peripheral blood T cell counts (lymphopenia) are observed in severe COVID-19 patients [6,13], with further reductions in those admitted to intensive care units (ICUs) and in those over the age of 60 [32], whereas increased SARS-CoV-2-specific T cells are associated with disease recovery [33,34,35,36]. There are three potential reasons for lymphopenia in severe COVID-19 patients. One is likely due to the SARS-CoV-2 spike proteins directly interacting with CD26 on T cells, leading to T cell apoptosis and immune dysfunction [37,38]. The second is due to the relocation of T cells, assuming that a large number of T cells in the blood are recruited to the lung [15,39]. Additionally, the third, seen in aged patients, is possibly attributed to the aged patient’s low thymopoiesis [40,41], which in conjunction with immunosenescence, reduces efficient peripheral T cell activation and differentiation for the necessary anti-infection response [42].

The exact roles of the aged T cell system in the clinical severity of COVID-19 disease remains unclear, but there are at least three considerations, which can all be traced back to the aged, atrophied thymus, and the consequences of immunosenescence and inflammation. First, immunosenescence (reduced immune responsiveness) in the T cell system is attributed to both decreased output of functional naïve T cells and accumulated exhausted/senescent memory T cells in the periphery, and restricts overall TCR diversity [43,44]. Second, immunosuppression from enhanced and accumulated polyclonal Treg cells, which serve the vital function of suppressing excess immune responses mediated by effector T (Teff) cells and other immune cells both with and without antigen-specificity (polyclonal Treg cells can exert bystander suppressive effects), serves to maintain immunological self-tolerance. In aged individuals, however, abnormally accumulated peripheral regulatory T (pTreg) cells may negatively impact anti-infection responses and vaccination. Third, inflammaging, which is partially attributed to increased self-reactive T cell output, could exacerbate COVID-19 pathology and possibly inhibit T cell responses to vaccination [3].

In addition, many uninfected healthy people were reported to have pre-existing SARS-CoV-2-specific T cells, possibly due to the cross-reactive memory T cells induced by previous infection with coronaviruses of the common cold, and these individuals seem less susceptible to SARS-CoV-2 infection [33,34,35,36]. This confirms the critical function of T cells in anti-SARS-CoV-2 immunity. The pre-existing common cold-specific memory T cells in the elderly could be exhausted and/or senescent, which is another reason that the aged people cannot adapt to new infection. Thus, it is reasonable to speculate that for these reasons, aged people are highly susceptible to severe SARS-CoV-2 cases with a poor prognosis, and may experience lower efficacy with COVID-19 vaccines, compared to young adults.

## 3. How Does Age-Related Thymic Involution and Subsequent T-cell Alterations Contribute to Severity of COVID-19 Pathophysiology in the Elderly?

Age-related thymic involution alters T cell profiles in ways that compromise immune function exhibited by several obvious characteristics, the first of which is reduced output of functional naïve T cells [30,45,46,47], which, coupled with accumulated exhausted/senescent memory T cells, results in a restricted TCR repertoire diversity, and contributes to immunosenescence, i.e., cellular immune functional insufficiency [48]. The second is increased output of self-reactive T cells, resulting in increased self-reactivity [49], involved in inflammaging, i.e., enhanced basal inflammation in the elderly [50,51,52]. Although seemingly opposing functions, these two phenotypes are interconnected [16,53]. The third is relatively enhanced polyclonal thymic regulatory T cell (tTreg) generation via an increased ratio of newly generated tTreg cells to thymic T conventional (tTcon) cells [54], which potentially exacerbates the age-related accumulation of pTreg cells [55,56,57,58]. The outcome of excess pTreg cells in the elderly is likely a disruption of immune homeostasis or imbalanced responses against foreign antigen and/or suppression of self-antigen-directed responses. Herein, we suggest that the impacts of these alterations in the aged T cell system, associated with age-related thymic involution, are potentially involved in the clinical severity of COVID-19 infection in elderly patients.

In addition to the restricted TCR diversity, which limits the ability of the aged T cell system to respond to novel pathogens, including SARS-CoV-2 [18], immunosenescence, characterized by reduced T cell response in the elderly, is also a major defect in aged antiviral immunity. Specifically, elderly individuals have accumulated CD28^neg-^ T cells, which cannot receive the necessary secondary T cell activation signaling [59,60,61], and exhibit multiple senescent markers, such as programmed cell death protein 1 (PD-1) [62,63] and p16(INK4a) [64,65,66]. Therefore, these senescent T cells (CD28^-neg^ and/or PD-1^+^ CD8^SP^ and CD4^SP^) dampen the normal T cell response to specific antigens. Importantly, these accumulated senescent T cells can also express the nature killer receptor (NKR). NKR^+^ T cells act as NK cells and can kill cells of various tissues that express NKR ligands during inflammation. Accumulated senescent T cells can infiltrate into various tissues including the lung, in older individuals. Therefore, if these aged T cells enter the lungs of older COVID-19 patients, they can induce inflammation via NKR without prior antigen-specific priming.

Increased output of self-reactive T cells from the aged, atrophied thymus results from perturbation of thymocyte negative selection [49,67]. These self-reactive T cells potentially participate in inflammaging, by infiltrating into non-lymphoid tissues and inducing self-tissue damage. This is concomitant with the previously defined chronic activation of innate immune cells in the elderly, which in conjunction with somatic cellular senescence produced SASP, results in increased circulating pro-inflammatory cytokines, characterized by above baseline serum concentrations of C-reactive protein (CRP), TNF-α, IL-6, and IL-8, in the elderly [68,69,70]. Inflammaging could exacerbate COVID-19 pathology and might even inhibit T cell responses to SARS-CoV-2 vaccines [3], due to downregulating the expression of T cell co-stimulatory molecule CD28 [71,72]. This pre-existing inflammatory condition may also initiate an inflammatory cascade that results in hyper-inflammatory responses in the lung during SARS-CoV-2 infections in older patients [3]. We speculate that the increased basal levels of pro-inflammatory signals and sub-clinical self-tissue damage might predispose certain individuals to certain types of infections that merely exacerbate the underlying immuno-reactive microenvironment in those tissues, such as the lung in the case of COVID-19. Indeed, our investigations have shown that in mice with thymic involution, there was increased lymphocyte infiltration into self-tissues, including the lung [67]. Although there is increasing interest in the correlation between immunosenescence and the increased risk of COVID-19 mortality in the elderly, more research is needed to fully elucidate the role of pre-existing lung inflammation and infiltration of potentially self-reactive T cells during COVID-19 pathogenesis [73,74,75,76].

Treg cells play a vital function in suppressing excessive immune responses mediated by Teff cells and other immune cells (B, DCs, NK, etc.), both with and without antigen-specificity, in order to maintain immunological self-tolerance [77,78]. However, it is also well established that pTreg cells accumulate with age and this abnormal accumulation has been implicated in immunosuppression of anti-infection and anti-tumor immunity, and inhibition of vaccination efficacy in the elderly [56,57,79]. For example, (a) in chronic *Leishmania major* infection, old mice had a higher percentage of pTreg cells and a lower capacity to clear the infection, while Treg depletion in these old mice increased Teff function [80]. Thus, increased pTreg cells exhibit a blockade to effectively fighting infection [81]; (b) in anti-tumor immunity, tumor-infiltrating pTreg cells usually enhance the suppression of CD8-mediated anti-tumor immunity to facilitate tumor cell survival [82]; (c) Treg cells were shown to block immune responses to a DNA vaccine via suppression of NK cells at the site of inoculation [83]; (d) transiently inhibiting FoxP3 impairs Treg activity and enhances the immunogenicity of vaccines, which improves vaccination efficacy [84].

Studies on Treg cells in COVID-19 patients are insufficient, but some reports showed that Treg cells within peripheral blood mononuclear cells (PBMCs) of COVID-19 patients were decreased [10,11], while other reports found a relative increase in COVID-19 patients with severe disease or/and lymphopenia [12,13]. If the decreased Treg cells in PBMCs are due to the pulmonary recruitment of these cells along with Teff cells [15], which is one of the potential reasons for lymphopenia in severe COVID-19 patients [6], perhaps we should ask why aged patients do not have less lung inflammation compared to young COVID-19 patients, since those aged Treg cells have relatively enhanced suppression function [79].

Another report also demonstrates that higher proportion of Treg cells might be related to severe COVID-19 disease. When compared to adult patients, pediatric patients, who had shorter length of illness and mild symptoms, had lower antigen-reactive (SARS-CoV-2 spike protein) CD4^+^CD25^+^ T cells (Treg-enriched cells), but adult patients with severe disease had a higher proportion of these Treg-enriched cells [85]. A different study did not support either the observation of Treg cell reduction or increase in COVID-19 patients, since the report showed that absolute Treg cell numbers were unchanged in COVID-19 patient blood compared to healthy people, although the percentage of Treg cells was increased in COVID-19 patients [86]. These inconsistent reports regarding Treg cells in COVID-19 patients are complicated by the fact that Treg cell data were collected from PBMCs, but not from the lung, which is the critical site of strong inflammation during COVID-19 infection and would therefore need Treg cells to suppress excessive immune reaction and control severe COVID-19 symptoms [14]. In addition, currently, there are no reports outlining the functional profiles of Treg cells in aged COVID-19 patients, who actually have age-related accumulation of pTreg cells in the periphery prior to the infection.

## 4. How can We Sufficiently Restore Antiviral Immunity and Improve Vaccine Efficiency in the Elderly?

Currently there are several proposed immune interventions for rebooting anti-COVID-19 immunity mostly focused on enhancing T effector cell responses and ameliorating immune cell-induced cytokine storm [15,87], which is more deadly in the elderly. Given that there appears to be profound T cell dysfunction in severe, particularly in aged, COVID-19 cases [32,88,89], rebooting T cell function by restoring thymic function should be considered as a potential holistic treatment for improving antiviral immunity and vaccination efficiency and potentially improve COVID-19 prognosis [76]. Along with rejuvenation of aged thymic function, refreshing the peripheral senescent T cell system, enhancing immune homeostasis, and reducing chronic peripheral inflammation, is also important for boosting antiviral immunity and vaccination efficiency [3,17,18]. Therefore, combination strategies to rejuvenate multiple aging-impacted immune system checkpoints, including aged thymic function and the peripheral T cell pool, as well as age-related basal inflammation, should be more efficient for improving anti-SARS-CoV-2 immunity and vaccine efficacy in the elderly.

One of methods related to enhancing thymic function, which has been used in clinical trials for the treatment of aged and severe COVID-19 patients, is Tα1. Although the underlying mechanism of this treatment is unclear, Tα1 is a thymic epithelial cell (TEC)-derived polypeptide hormone, which effectively supports T cell generation, maturation, and survival [90,91,92]. A clinical trial demonstrated that Tα1 restored CD8^+^ and CD4^+^ T cell numbers in severe COVID-19 patients with lymphopenia and reversed the PD-1 and Tim-3 expression on exhausted/senescent CD8^+^ T cells. Thereby, the mortality was decreased by 60% in severe COVID-19 patients [21].

Currently there are multiple strategies for thymic rejuvenation, not only via fostering thymus regrowth, but also restoring thymic function to enhance negative selection and rebalance Treg cell generation. Although these rejuvenation outcomes cannot have immediate impacts on patients suffering from acute infection, using these therapeutic strategies in advance to holistically improve immune function in the elderly could significantly reduce their mortality and morbidity in this pandemic, as well as improve their vaccination efficiency. The most promising strategies for thymic rejuvenation include improvement of TEC homeostasis via *FOXN1* gene. *FOXN1* is a master transcription regulator for the growth and differentiation of TECs [93,94], and declined *FOXN1* gene expression contributes to age-related thymic atrophy [95,96]. Intrathymic injection of *FOXN1* reprogrammed embryonic fibroblasts cells significantly promoted regrowth of the aged, atrophied thymus and ameliorated T cell senescence-induced inflammaging in a mouse model [97]. Thymus transplantation is a compensatory strategy applied clinically to treat DiGeorge syndrome patients born without a functional thymus to accomplish T cell generation [98,99]. However, since the increased self-reactive T cells produced by the aged thymus cannot be inhibited, thymus transplantation cannot alleviate self-reactive T cell-induced inflammaging, which is a potent predisposition for inflammatory cytokine storm in the elderly.

Another, more clinically practical, approach for thymic rejuvenation is to use cytokines, growth factors, hormones, and other blood-borne factors. For example, a developed fusion protein that combined IL-7 and N-terminal extracellular domain of CCR9 to target the thymus of aged animals, restored thymic architecture and thymopoiesis [100]. IL-7 is a pleiotropic cytokine, essentially required for early thymocyte development [101,102] and lymphocyte survival and expansion [103,104], but its expression declines in the aged thymus [105]. IL-7 can also maintain the homeostasis of peripheral naive T cells and memory T cells [106], as well as enhance the activation of follicular T helper cells (Tfh) which interact with B cells in germinal centers for antibody production [107]. We noticed that recombination IL-7 was used in treatment of severe COVID-19 patients [108,109]. The outcomes showed a return of CD4+ and CD8+ T cell levels to a reference level [108], although the underlying mechanisms and clinical significance of this treatment are yet to be determined. Growth hormone (GH) has a role in thymic rejuvenation and promotes immune reconstitution by stimulating the production of insulin-like growth factor-1 (IGF-1), which acts on thymic stromal cells and stimulates IL-7 production [100]. It has been suggested to use GH to reduce the vulnerability of some at-risk groups of patients during this COVID-19 pandemic [110].

Many strategies can be used for peripheral T cell functional restoration. For example, senescent T cells have increased PD-1 expression, therefore, blocking PD-1 on CD4 and CD8 T cells with an anti-PD-1 antibody in aged individuals can partially restore the decreased production of IFN-γ [111]. Since elderly individuals have chronic inflammatory conditions, which can suppress immune responses and vaccination efficiency [112,113], reducing long-term self-reactivity-induced inflammaging, via suppressing mTOR (the mammalian target of rapamycin) is a promising strategy. The mTOR signaling pathway regulates various aspects of the immune response including T cell subset differentiation, function, and proliferation of Treg cells, and memory T cell generation [114]. Rapamycin, an mTOR inhibitor, has been shown to augment cell memory after vaccination [115]. A low dose combination of mTOR inhibitors RAD001 and BEZ235 enhanced antibody responses to influenza vaccination and reduces respiratory infection incidence in the elderly [116], which reveals a potential role of the mTOR signaling pathway in vaccination efficiency in the elderly [117]. The dosage should be one of the key considerations because the mTOR signaling activation is also involved in Th1 and Th17 subset differentiations [118,119,120]. Therefore, mTOR inhibitors are a potential immunoregulatory target during COVID-19 vaccination and treatment in the aged population.

The cytokine storm syndrome in COVID-19 patients is mainly characterized by the IL-1 family, IL-6, and TNF-α [121,122,123], among which the serum TNF-α level is negatively correlated with T cell function by downregulating the expression of co-stimulatory molecule CD28 [71,72]. Inhibition of TNF- α with antibody or a TNF-α receptor inhibitor delays the loss of CD28 expression on CD8 T cells during replicative senescence [124]. Likewise, TNF- α suppresses B cell immune responses [125,126] and B cells in aged individuals produce higher TNF- α than in young individuals [126]. Therefore, anti-inflammatory drugs, such as aspirin, could potentially restore adaptive immune response to COVID-19 in the elderly. In addition, aspirin was able to enhance IFN-γ production by Th1 cells [127], which may be favorable for antiviral immunity.

As discussed previously, accumulated CD4^+^Foxp3^+^ Treg cells during aging are probably a double-edged sword in SARS-CoV-2 infection. If there are too many Treg cells, the antiviral immunity and vaccination efficiency mediated by effector T cells and B cells may be suppressed, resulting in reduced inhibition of viral replication in the elderly; whereas, if Treg cells are insufficient in the inflammatory lung, the excessive immune reaction-induced tissue damage could be detrimental. Utilizing anti-CD25 to block Treg cell function has been demonstrated to augment protective immune responses to influenza virus-like particles in aged mice [128]. Induced Treg (iTreg) cells can be generated via transforming growth factor- β (TGF-β), while blocking TGF-β signaling impedes the conversion of CD4 T cells into iTreg cells and thereby facilitates immune responses abrogated by Treg suppression [129]. This explains the recent suggestion to use TGF-β blockade to treat COVID-19 patients [130]. The underlying mechanism is likely related to inhibiting iTreg generation and suppressing lung fibrosis induced by TGF-β during severe COVID-19 cases [130].

Blood-borne extracellular vesicles (EVs) from young blood cells, containing exosomes encapsulating many regulatory signaling molecules, such as mRNA, microRNA, DNA and proteins, are another promising rejuvenation reagent to regulate the aged immune system [131]. In our previous research, serum-derived EVs isolated from young mice and administered to aged mice were able to partially restore thymocyte negative selection and alleviated systemic inflammaging in the periphery of age mice [132]. Therefore, rejuvenation of the aged immune system via young serum-derived EVs is an example of a combinational rejuvenation strategy.

Comprehensive strategies to rejuvenate multiple aging-impacted immune system checkpoints, not only the thymus, but also peripheral T cell profiles, are holistic and potentially more effective than single treatments. Although comprehensive rejuvenation strategies are at the early proposal stage, we speculate a promising strategy targeting multiple aging-impacted immune system checkpoints (Figure 1B and Table 1 rightmost column), based on our previous experience and current literature. In combination with aged thymus rejuvenation, such as via reprogrammed *FOXN1*-expressing fibroblasts [97], the peripheral rejuvenation should focus on reducing inflammation and restoring T cell homeostasis.

Taken together, based on current evidence, modulating the central and peripheral T cell immune system is a promising therapeutic strategy for COVID-19 in the elderly. However, comprehensive clinical trials remain to be performed to evaluate the effectiveness and safety of these methods in the case of COVID-19.

## 5. Concluding Remarks

Although it has become increasingly clear that T cells play a central role in generating powerful and long-term immunity and clearance of SARS-CoV-2 infection, the synergistic effects of immunosenescence and inflammaging associated with thymic aging remain to be elucidated. Mounting evidence shows that a proportion of pre-existing SARS-CoV-2-specific T cells may have arisen from a previous infection with common cold coronaviruses, which may play a protective role against SARS-CoV-2 infection-induced severe symptoms [33,34,36,133]. However, there is insufficient research about whether aged individuals have the same proportion of these cross-reactive T cells as young individuals, and whether these cross-reactive T cells can exert the same level of protection in the elderly, who have underlying impacts of age-related thymic involution, immunosenescence, and inflammaging. In addition, it is urgent to study which types of SARS-CoV-2 vaccines are more effective in the elderly who have aged T cell immunity and reduced naïve T cells. There is a need for a deeper understanding of how the aged thymus, and subsequently altered aged T cell system, impacts SARS-CoV-2 infection in the elderly. Finally, investigating how to improve these aspects of detrimental immune dysfunction in the elderly will reveal how to generate more robust immunity to COVID-19 and reduce their high morbidity and mortality during COVID-19 infection.

## Figures and Tables

**Table 1:** Contributions of aged thymus to viral infection and potential rejuvenation therapeutics.

	Normal Thymus Maintains Homeostasis and Immunity	Age-related Thymic Changes Contribute to Viral Infection	Potential Rejuvenation Strategies
Thymus	1. Sufficient naïve T cell generation with highly diverse TCR repertoire 2. Minimal self-reactive T cell generation 3. tTreg generation balanced with tTcon generation	1. Reduced functional naïve T cells 2. Increased self-reactive T cells 3. Enhanced tTreg generation in proportion to tTcon output	Thymic rejuvenation via: 1. Injecting reprogrammed *FoxN1* over-expressing fibroblasts 2. Providing exogenous factors such as growth hormone, IL-7, etc.
Peripheral lymphoid tissues and circulating blood	1. T cells with normal TCR repertoire → a broad recognition of foreign antigens 2. Potent T cell immune response to foreign antigens and homeostatic clearance of senescent somatic cells 3. pTreg cells balanced with pTcon cells → maintenance of immune tolerance and antiviral immunity.	**1. Immunosenescence:** Restricted TCR repertoire diversity → compromised viral antigen recognition Accumulated exhausted T cells → compromised anti-viral immune response and senescent somatic cell clearance → inflammaging Accumulated pTreg → suppress normal antiviral immune responses **2. Inflammaging:** Self-reactive T cell induced tissue damage → chronic basal inflammation → inhibition of T and B cell activation for antiviral responses	1. Enhance peripheral T cell function via: a. TGF-β blockade to inhibit iTreg cells b. PD-1 blockade 2. Reduce chronic inflammatory conditions via low-dose mTOR inhibitors, aspirin, etc.
Lung	1. Sufficient cellular and humoral antiviral immunity 2. Timely clearance of virus by appropriate pro-inflammatory responses	1. Reduced antiviral function by T cells and plasma cells 2. Inflammatory cytokine storm facilitated by inflammaging 3. Lung tissue fibrosis after inflammation	TGF-β blockade to reduce fibrosis

Abbreviations: IL-7: interleukin-7; iTreg: induced T regulatory cells; TCR: T cell receptor; TGF-β: Transforming growth factor-β; tTcon: thymic conventional T cells; tTreg: thymic regulatory T cells; mTOR: mammalian target of rapamycin; PD-1: Programmed cell death protein-1.

## Data Availability

Not applicable.

## Highlights

1. COVID-19 exhibits severe symptoms and high mortality in elderly individuals. 2. T cell immunity in COVID-19 plays an important role, while the aged T cell system contains both immunosenescence and inflammaging. 3. These changes can be traced back to age-related thymic involution, which is an obvious difference between aged and young individuals. 4. How the aged thymus is involved in impacting viral infection and how a proposed comprehensive rejuvenation strategy works in antiviral immunity and vaccination efficiency are addressed.

## Abbreviations

Ab	antibody
COVID-19	coronavirus disease 2019
DC	dendritic cell
EVs	extracellular vesicles
GH	growth hormone
iTreg	induced regulatory T (Treg) cell
IFN-γ	interferon-γ
NK	nature killer cell
NKR	nature killer receptor
mTOR	mammalian target of rapamycin
PBMCs	peripheral blood mononuclear cells
pTreg	peripheral regulatory T (Treg) cell
PD-1	programmed cell death protein 1
SASP	senescence-associated secretory phenotype
SARS-CoV-2	severe acute respiratory syndrome coronavirus 2
TCR	T cell receptor
Teff	effector T cell
TEC	thymic epithelial cell
tTreg	thymic regulatory T (Treg) cell
Tα1	thymosin alpha-1
TGF-β	transforming growth factor-β
TNF-α	tumor necrosis factor-α
